# Potential Application of Epoxy Powder Coating Waste in Concrete: Strength Properties and Durability of Concrete

**DOI:** 10.3390/ma19091756

**Published:** 2026-04-25

**Authors:** Janusz Konkol, Bernardeta Dębska, Andriy Huts, Barbara Pilch-Pitera, Guilherme Jorge Brigolini Silva, Cristopher Antonio Martins De Moura, Wioleta Iskra-Kozak, Jerzy Szyszka

**Affiliations:** 1 Faculty of Civil and Environmental Engineering and Architecture, Rzeszow University of Technology, 35-029 Rzeszow, Poland; janusz.konkol@prz.edu.pl (J.K.); bdebska@prz.edu.pl (B.D.); w_iskra@prz.edu.pl (W.I.-K.); jszyszka@prz.edu.pl (J.S.); 2 Faculty of Chemistry, Rzeszow University of Technology, 35-029 Rzeszow, Poland; 3 Departamento de Engenharia Civil, Universidade Federal de Ouro Preto, Campus Morro do Cruzeiro, Ouro Preto 35400-000, MG, Brazil; guilhermebrigolini@ufop.edu.br; 4 Instituto de Ciências Exatas e da Terra, Universidade Federal de Mato Grosso, Campus Universitário do Araguaia, Barra do Garças 78060-900, MG, Brazil

**Keywords:** sustainable concrete, circular economy, epoxy powder coating waste, compressive strength, durability of concrete

## Abstract

This paper presents the results of tests on concrete modified with waste powder from the production of epoxy powder coating, planned using design of experiment’s (DOE) experimental design methods. The scope of the investigation included detailed identification of the waste itself (TG/DTA, FTIR, SEM + EDS, laser diffraction), as well as evaluation of selected properties of concretes containing this waste, including compressive strength, density, and durability parameters such as frost resistance and chemical resistance. The scope of the experiment was defined by varying modifier content in the range of 4 to 11% of the cement mass and a water-cement ratio between 0.44 and 0.56. The concrete mixes obtained were characterized by good workability, fluidity, and consistency stability over time, despite the use of the modifier as an additional component in the concrete mix. No adverse effect of the waste used on the durability of the concrete was observed. Concretes modified with waste from the production of epoxy powder coating achieved a frost resistance class of F150 and showed good resistance to chemically aggressive environments (sulfates and chlorides). No products indicating adverse reactions between waste powder and reagents were found. The use of the DOE approach made it possible to determine, in the form of functional relationships, the influence of the modifier content depending on the water-cement ratio (*w*/*c*) of the concrete on its compressive strength and density. In general, a decrease in the compressive strength of concrete containing a waste powder modifier was observed, ranging from approximately 11% to 26% compared to unmodified concrete. However, the trend of decreasing compressive strength was reduced as the water-cement ratio of concrete decreased. At a water-cement ratio (*w*/*c*) of 0.443, no further decrease in compressive strength was observed. Concrete with 11% waste powder and a *w*/*c* ratio of 0.443 achieved 4.7% higher compressive strength than unmodified concrete with the same water-cement ratio. A beneficial interaction was found between a carboxylate-based plasticizer and the waste powder from the production of epoxy powder coatings. The proposed method of using waste as a concrete component is promising and may contribute to reducing the problem of waste management, as well as greenhouse gas emissions.

## 1. Introduction

In recent years, the development of technologies related to the use of industrial waste in building materials has become an important area of research aimed at reducing the negative impact of this waste on the environment. There is a series of publications that point to the benefits of adding various types of waste to the widely studied properties of cement matrix composites. However, the use of waste from powder coating production in concrete manufacturing is an area that has not yet been sufficiently explored. This type of coating accounts for a significant share of the paint market, and its production is continuously growing [1,2], generating large amounts of powder waste, including unused overspray.

Depending on the decorative and technical requirements are based in approximately 60–65% on polyester resin, epoxy resin, epoxy-polyester mixtures, or less often on acrylic resin, and in the case of extremely high resistance to weather conditions, such as fluoropolymers [3,4]. Polyester, polyurethane, or polyacrylic coatings contain approximately 5% hardener, which reacts with the resin at 160–180 °C. Barite and calcite act as fillers and constitute approximately 20–25% of the powder coating. The remaining components are pigments (4–8%), flow control, and degassing agents (1.5–2%). The particle sizes of the powder coating are typically in the range of 1 to 120 µm. Powder coating waste is recycled by reusing it in the painting process. However, the fraction of ultrafine particles below 10 µm, captured by separation (cyclone) and filtration system, is not suitable for reuse in the powder coating process due to its poor ability to charge electrically. This fraction is then recycled in other areas or disposed of.

Conventional methods for utilizing this type of waste, such as incineration, are undesirable. Storage in the form of waste piles also poses a significant environmental burden. Therefore, the search for technologies that allow the reuse of this material in the production cycle is an important area of research that fits the principles of the circular economy. Demonstrating the possibility of using this type of waste in cement matrix composites offers great opportunities both to reduce the environmental impact by reducing the amount of waste going to landfills, as well as to reduce CO_2_ emissions and, importantly, to limit the consumption of mineral resources.

The use of polymers in cement composites, either as modifiers or as partial binders, has been widely studied. However, it should be noted that polymers may play different roles depending on their form and function. Polymer-based admixtures, such as plasticizers (e.g., polycarboxylate ethers), primarily act as dispersing agents, improving the workability and rheological behavior of fresh mixtures by reducing the interparticle attraction and water demand. In contrast, polymer modifiers such as latexes, redispersible polymer powders, or liquid resins form a continuous or semicontinuous organic phase within the cement matrix, which contributes to microstructural refinement. As a result, polymer modification can lead to improved durability, including enhanced resistance to chemical aggression, reduced permeability, and increased adhesion. Additionally, the presence of a polymer phase may increase deformability and tensile strength. Polymer cement concretes (PCC) [5,6,7] and polymer concretes (PC) [8] are well-established materials and remain an important area of research aimed at improving the durability and performance of cement-based composites. As noted by Salami et al. [8], polymers are commonly used in concrete technology in various forms, including admixtures (e.g., superplasticizers), fibers, and redispersible powders, each fulfilling distinct functions in the composite system.

The epoxy polymer, which belongs to the group of thermosetting polymers, which together with thermoplastic polymers and elastomers, constitute one of the three categories of polymers used, can be used as a modifier of the composition of PCC concrete. However, the curing mechanism and interactions between cement hydration products and the polymer will be different if the waste from the production of epoxy powder coatings is used as a modifier. In general, particles of this type of waste will act as an inert component in concrete, although due to their size, they will also seal the microstructure of the hardened cement paste. Uncured or partially cured epoxy resin, which constitutes a significant proportion of the waste, will not have suitable conditions for complete and controlled epoxy cross-linking. Therefore, only a few epoxy groups can be opened in the strongly alkaline environment of the cement paste and partially bond to the cement matrix. The remaining parts of the waste will act as filler. An important issue, which is also the subject of this manuscript, is the impact of other waste components, including, in addition to epoxy resin, calcite and barite, as well as hardeners, which account for a relatively large proportion of the waste volume. Some researchers have shown that additives of this type can be a valuable modifier of concrete, also as a substitute for cement (barite, hematite, and lead powder [9], calcite-waste toner powder [10]).

From an environmental perspective, incorporating waste from the production of epoxy powder coatings into cement matrix composites can involve three issues: reducing the environmental impact by decreasing the amount of waste sent to landfills, limiting the consumption of natural raw materials (the waste is used as a filler), and reducing the carbon impact associated with the waste disposal process.

The addition of waste as a recyclable material to the composition of the concrete mixture is also a health-promoting measure, limiting the potential adverse effects of direct contact with such waste. The main risk associated with direct contact with powder coating waste is serious respiratory disease or allergies, skin or eye irritation by small paint particles. Therefore, introducing this waste as a concrete component and following the typical safety rules of concrete plants during production, as is the case with silica fume, eliminates the risk to human health. They are also nontoxic substances.

The use of resin-rich waste in the form of finely ground powder may act as an inert microfiller in cement composites. Due to the typically low chemical reactivity of such materials with the cement matrix, their effect is primarily physical rather than chemical. In particular, fine particles can fill voids between cement grains, leading to denser particle packing and a refinement of the pore structure. This may result in reduced permeability and limited transport of water and aggressive agents, which are known to influence the durability of cement-based materials. However, the effectiveness of this mechanism depends on factors such as particle size distribution, morphology, and the level of dispersion within the matrix.

Previous studies have shown that adding polymer in the form of powder coating waste to concrete acts as a plasticizer in the concrete mix [11]. The powder coating waste used in these studies contained 56% polyester resin. The study [11] found that the use of powder coating waste as a pigment material to color silicate bricks was not very effective. However, it was pointed out that the use of waste in an amount of 5% of the cement mass improved the fluidity of the concrete mix compared to a mix without this modifier.

Research on the use of waste from paint production also concerns the use of polymer emulsions used in emulsion paints, such as polyvinyl acetate [12,13], or waste from paint production in the form of wastewater and sewage sludge (for example, from the production of latex paints [14,15,16]).

Noruzman [12] conducted research on concrete modified with polyvinyl acetate derived from paint and adhesive industry waste [12]. A positive effect was demonstrated on the workability of the concrete mixture, as well as on the set time of the concrete and the heat of hydration, with the optimum compressive strength achieved at a modifier content of 2–3% by weight of cement. An increase in tensile and flexural strength was observed. The highest values of these parameters were obtained with modifications of 5% and 1% of waste relative to the weight of cement, respectively. A negative effect of using this modifier was the low resistance to chemically aggressive environments.

The impact of polymer as a material derived from latex paint waste was studied by Ismail et al. [16]. In this case, the effect of polymer on the strength and durability of the concrete was studied from 7 to 60 days of curing. A positive effect of the modifier on the strength properties was observed (indirect tensile and flexural strengths) and the durability of the concrete (resistance to chloride aggression), although increasing the polymer content to 10% of the cement weight had a negative effect on the consistency of the concrete mix and the compressive strength of the concrete. Assaad [14] investigated the results of rheological tests of modified cement paste with latex waste coatings. Paints were used as modifiers only after their expiration date and stored under various conditions. Differences in the behavior of cement pastes were observed in terms of their rheological properties (flow, viscosity) and compressive strength. An unexpected negative effect was an increase in the porosity of the cement stone.

Waste epoxy powder coatings also contain the hardener necessary for crosslinking the resin. However, this process can only take place under appropriate temperature conditions (160–200 °C). Therefore, polymerization resulting from the reaction with the hardener will not be possible in concrete. The curing agent derived from the powder coating (e.g., dicyandiamide, amine-epoxy adducts, and poliphenolic curing agents) will remain as an inactive phase. Hydration of alkaline phases, mainly alite and belite, in fresh cement paste results in the formation of a pore solution characterized by very high alkalinity (pH 13.0–13.8). The dominant alkaline components are sodium and potassium hydroxides, whose concentration increases rapidly in the first hours of hydration. This creates an environment rich in strongly nucleophilic compounds, primarily hydroxyl ions OH^−^. Under such conditions, epoxy resins added to the cement matrix undergo ring-opening reactions catalyzed by anionic components of the environment. This process occurs in parallel with clinker hydration and leads to the formation of products, but with a structure different from that obtained as a result of curing classic amine-epoxy systems. At the same time, local reactions of epoxy ring opening cause partial and heterogeneous crosslinking, and a significant part of the organic phase remains in the microstructure of the grout only as a filler.

Jo and Do [17] conducted research on the polymerization of epoxy resin in a strongly alkaline environment without the use of a hardener. They analyzed the curing properties of cement composites modified with epoxy resin without the addition of a hardener. Hydroxide ions formed as a result of cement hydration have been shown to catalyze the epoxy ring opening reaction, leading to partial curing of the resin and the formation of a three-dimensional cross-linked structure. The degree of curing of epoxy is strongly dependent on the polymer/cement ratio. With a polymer content of 10% by weight of cement, the highest degree of curing was achieved, ranging from 60 to 70%, while for values greater than 20%, a decrease was observed below 50%. This was confirmed by extraction methods, IR spectroscopy, GPC, TG-DTA, PXRD X-ray diffraction, and SEM observation. The results obtained indicate that with a polymer/cement ratio in the range of 10–20%, it is possible to obtain composites with positive mechanical properties without the need for a hardener. At the same time, it was found that excess uncured resin can act as a protective layer, limiting drying shrinkage and the penetration of water and salt.

The powder coating waste is an interesting material for use as an additive in concrete mixtures. It constitutes only a small percent of the total powder coating production, but because of its compliance with environmental requirements, unlike liquid paints, its production is rapidly increasing. The storage or burning of waste is very expensive and poses a burden to the environment. Therefore, the development of more ecological solutions is crucial. Due to the lack of toxic substances in their chemical composition and the possibility of favorable interactions between reactive functional groups present in resins, a raw material can be a highly valuable additive for use in concrete modification.

No reports in the literature have been found on the use of waste from the production of epoxy resin-based powder coatings containing 50–70% epoxy resin and hardener such as modified dicyanodiamide, carboylic acid, or polyphenol and fillers in the form of calcium carbonate and barite, which together with resin constitute 90–95% of the total composition in concrete.

The aim of this study is to investigate the effect of incorporating waste from the production of epoxy powder coatings on the properties of concrete and to assess its potential use as a modifier of the concrete composition. An important aspect of the work is the utilization of waste powder material in cement-based composites, which may contribute to reducing the amount of industrial waste directed to landfills through reuse in construction materials. Furthermore, partial replacement of conventional components can reduce the overall environmental impact of the material, including potential reductions in CO_2_ emissions associated with the production of raw materials. The results obtained enable a quantitative evaluation of the influence of this waste on selected physical and mechanical properties of cement composites, supporting its potential application as a functional modifier in construction materials.

## 2. Materials and Methods

### 2.1. Materials

#### 2.1.1. Cement

The test samples were made of concrete using Portland cement CEM I 42.5 R produced by Ożarów Cement S.A. (Ożarów, Poland) with properties in accordance with PN-EN 197-1 [18] and the following mineral composition: C_3_S—59.01%; C_2_S—13.68%; C_3_A—7.49%; C_4_AF—6.36%; and specific surface area 3.739 cm^2^/g. Other chemical and mechanical parameters of cement are presented in Table 1.

#### 2.1.2. Aggregate

Quartz sand of 0–2 mm fraction, produced by Kruszgeo S.A., Mrowla, Poland, and coarse basalt aggregate of 4–16 mm fraction, obtained from the Wilków-Bazalt S.A. quarry, Złotoryja, Poland, was used. Both quartz sand and basalt aggregate comply with the requirements of PN-EN 12620+A1:2010 [19].

#### 2.1.3. Plasticizer

The MasterGlenium ACE 560 plasticizer was selected as a water reducer according to PN-EN 934-2+A1:2012 [20]—a highly effective liquefaction admixture based on polycarboxylate ether technology (PCE—polymer consisting of segments containing carboxyl groups and polyether segments), produced by ‘Master Builders Solution’, Myslenice, Poland.

#### 2.1.4. Water

Tap water meeting the requirements of PN-EN 1008:2004 [21].

#### 2.1.5. Waste Powder

**Figure 1:**
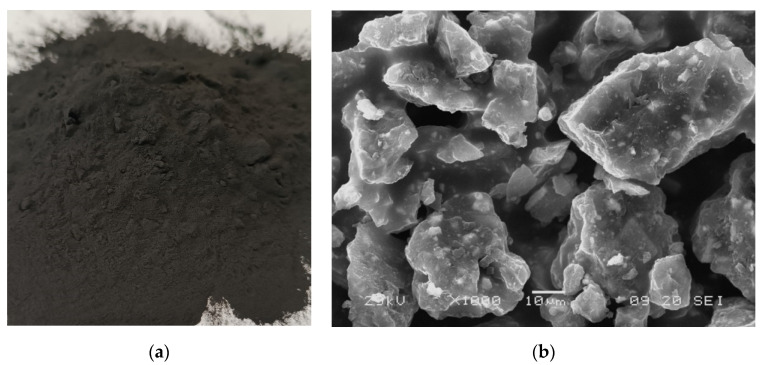
Waste powder used in research: (**a**) general view; (**b**) morphology of waste grains with a lamellar structure.

Waste from the production of epoxy powder coatings, supplied by Inver Poland sp. z o.o. (Dębica, Poland), was used as a concrete composition modifier. A detailed analysis of the characteristics of the supplied materials was carried out. SEM analysis of waste particle morphology revealed an irregular grain geometry (Figure 1), which is also characteristic of coarse crushed aggregate grains.

**Figure 2:**
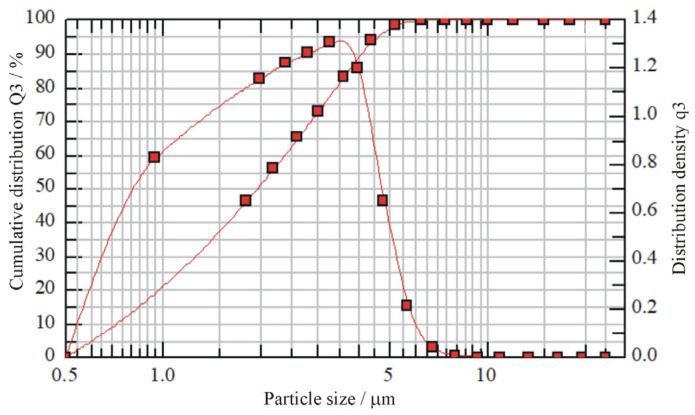
Particle size distribution of epoxy powder coating production waste (results provided by Inver Poland, Dębica, Poland).

The particle size distribution of the coating production waste measured by laser diffraction on a HELOS (H3493) and RODOS device is shown in Figure 2. Ninety percent of the particles fell within the size range of up to 5 µm, as illustrated by the cumulative distribution (Q3). The distribution density graph (q3) also shows the monodisperse nature of the particle size and allows the identification of the dominant fraction in the waste material (approximately 3–4 µm). Distribution density values reaching 1.4 also indicate high uniformity of particle size, which is essential information for analyzing the potential application of this additive in terms of its ability to fill the spaces between the particles of the other concrete components. The density of the waste was determined to be 1.71 g/cm^3^.

Due to the size and shape of the waste grains, it can be expected that it will have not only a sealing effect of the cement composite microstructure and the ITZ transition zone, but also an improvement in the quality of bonding between the grains with the cement matrix. This is due to better mechanical anchoring of the additive particles in the matrix and increased contact surface between the waste grains and the cement hydration products. The relatively high specific surface area of the waste (20,538 cm^2^/g) may also influence the rheological properties of the cement paste, particularly by increasing water demand and affecting viscosity and fluidity of the cement paste.

**Figure 3:**
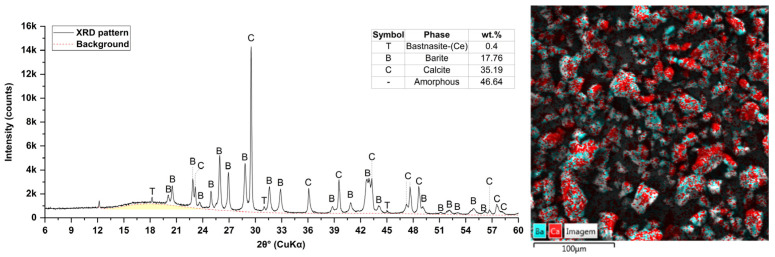
Results of the X-ray diffraction and SEM + EDS analysis of the epoxy waste powder.

The morphological SEM analysis shows a predominance of equidimensional and angular-shaped particles (Figure 1). Furthermore, the EDS mapping indicates the presence of elements Ba, Ca, and S, which is consistent with the XRD and XRF results (Figure 3). Qualitative XRD analysis shows peaks associated with barite, bastnasite, and calcite, as well as an amorphous halo between 12° and 24° (2θ). The qualitative composition of the XRF is as follows: Ce, Ca, S, Ba, Pr, Cu, Sr, Mn, Si, and Co.

### 2.2. Research Methods

Physical and mechanical methods were used in the experiments to study the main properties of concrete mixtures and concrete according to the requirements of current norms and standards. The experimental research program included the following stages:

#### 2.2.1. Sample Preparation

Basic concrete mixes and mixes with the addition of production waste from epoxy powder coating were prepared using strictly defined proportions of cement, coarse aggregate, sand, water, and modifier according to the adopted central composite design (CCD) experiment plan. The mixing process was carried out in a laboratory mixer according to the applicable standards. The concrete mixture was formed into appropriate molds according to [22]. Immediately after unmolding, the samples were placed in a climate chamber with a temperature of 20 ± 2 °C and a humidity of 95 ± 5%, where they were cured until the appropriate study time according to [23].

For thermogravimetry (TG) and differential thermal analysis (DTA), X-ray diffraction (XRD), and Fourier transform infrared spectroscopy (FTIR) analyses, concrete samples were crushed and manually milled in a mortar and then sieved using a 53-micrometer standardized sieve. For SEM analysis, the concrete samples were crushed and mounted on carbon tape attached to a metallic stub, followed by gold coating.

#### 2.2.2. Chemical Analysis and Microstructural Studies

For Fourier transform infrared spectroscopy analysis (FTIR), a Nicolet iS5 spectrometer (Thermo Scientific, Waltham, MA, USA) was used in the range of 4000 to 400 cm^−1^, with measurements performed on compressed tablets produced by mixing the sample of interest with KBr. The interaction between the epoxy resin and the plasticizer was carried out using the ATR technique.

Thermogravimetry and differential thermal analysis (TG/DTA) was carried out using a Mettler Toledo TGA/DSC 1 Stare System thermogravimetric analyzer (Mettler Toledo, Columbus, OH, USA), under standardized measurement conditions with a temperature range of 30 °C to 980 °C, a heating rate of 10 °C/min, and an N_2_ gas flow of 30 mL/min. Concrete samples were analyzed in a closed alumina crucible, while the waste sample was analyzed in an open alumina crucible. In addition, the thermal properties of the powder coating waste were analyzed using a Mettler Toledo type 822e differential scanning calorimeter (DSC) (Mettler Toledo, Columbus, OH, USA), operated with Stare System 16.20 software. Aluminum crucibles containing samples weighing 0.015 g were placed in the measurement chamber. Measurements were made in a nitrogen atmosphere at a flow rate of 60 cm^3^/min, covering a temperature range from 0 to 220 °C. The heating was carried out at a rate of 10 °C/min.

For X-ray diffraction (XRD), a Bruker D2 Phaser diffractometer was used for mineralogical prospecting, operating at 10 mA and 30 kV. Concrete samples and waste samples were analyzed under standardized measurement conditions using a backloading preparation method, with a scanning range of 5° ≤ 2θ ≤ 60°, a step size of 0.01819°, and 1.25 s per step. Qualitative phase identification was carried out based on reference patterns from the Crystallography Open Database (COD) [24,25], using X’Pert HighScore Plus software version 5.3a.

The SEM testing was performed using the Tescan Vega 3 scanning electron microscope (Tescan, Brno, Czech Republic). The observations were made on waste powder material and on the microstructure of hardened concrete modified with waste powder from paint production. The JSM–5500 LV microscope (manufactured by “JEOL”, Tokyo, Japan) was also used for this study.

#### 2.2.3. Consistency of Fresh Concrete Mixture

The consistency of the concrete mix was tested using the flow table method according to the standard [26]. The consistency measure of the mixture is the flow value, determined as the average value of the diameter of the flow of the mixture on the table in two perpendicular directions.

#### 2.2.4. Compressive Strength of Concrete

The compressive strength at the age of 28 and 90 days was performed on a FormTest PRÜFSYSTEME press, type ALPHA 3-3000S, manufactured by “Form + Test Seidner & Co. GmbH”, Riedlingen, Germany, according to [27]. For the test, six 10 cm × 10 cm × 10 cm samples were made for each concrete mixture.

#### 2.2.5. Density of Concrete

The density of concrete was determined using a geometric method in accordance with the requirements of PN-EN 12390-7 [28]. The test was carried out on samples cured under high humidity conditions (>95%) for 28 days. Then, the actual dimensions of the samples were measured accurately to within 0.1 mm and their weight was measured accurately (to within 0.1% of the sample weight). The average density of concrete ρ was determined based on the results of six samples measuring 10 cm × 10 cm × 10 cm for each series.

#### 2.2.6. Concrete Frost Resistance

Concrete frost resistance was tested using the standard method according to PN-B-06265 [29] on the Toropol K-015 refrigerator, manufactured by Toropol Sp. z o.o., Warsaw, Poland. Freeze–thaw cycles consist of successive freezing of the entire sample in air and unfreezing it in water, and the duration of a complete cycle is at least 6 h. Freezing is carried out in air at −18 ± 2 °C and lasts at least 4 h. Defrosting of the samples in water at +18 ± 2 °C—time of 2–4 h. After the last freeze–thaw cycle, the samples are weighed and the compressive strength is tested. The standard method for testing the frost resistance of concrete is based on three criteria for assessing frost resistance, such as the decrease in compressive strength of samples after a given number of freeze/thaw cycles and thawing cycles in relation to samples kept in water throughout the entire period, the loss of mass of the samples after freezing and thawing cycles and the occurrence of any microcracks. The test is carried out on 12 cubic samples; six subjected to freeze/thaw cycles and six kept throughout the test in water at a temperature of +18 ± 2 °C. The 12 samples were previously saturated with water for at least 7 days.

#### 2.2.7. Research Plan

In order to program the tests, the theory of experimental design was used, supplemented by a comprehensive statistical analysis of the test results obtained. Both the experiment planning stage and the analysis of the results were carried out using the Statistica 13 software.

**Figure 4:**
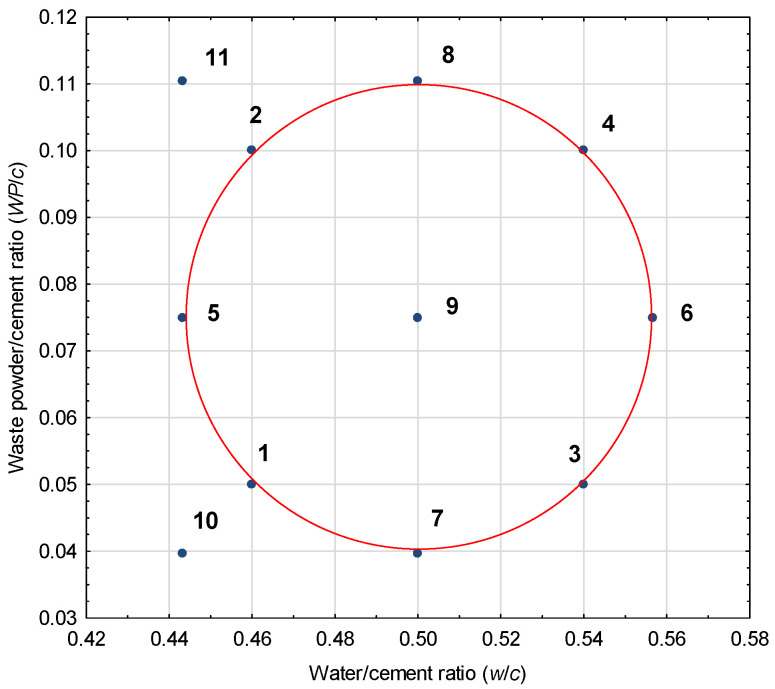
Arrangement of research points in the experimental design.

In terms of strength testing, a composite central experimental design with two input variables was adopted. The input variables (independent variables) were the water/cement ratio (*w*/*c*) and the proportion of waste from the production of epoxy powder coatings relative to the mass of cement (waste powder/cement; *WP*/*c*). The number of test points for the adopted plan was 9. Furthermore, tests were carried out on modified concretes with low *w*/*c* ratios and unmodified concretes with varying water-cement ratios. The adoption of additional test points was designed to increase the scope of the experiment in view of the needs of local concrete producers. The adopted test point layout is shown in Figure 4. The range of variation in the *w*/*c* and *WP*/*c* variables, which determine the composition of the concrete mixture, was established on the basis of preliminary laboratory tests on trial mixes.

Concrete mixes were prepared with a variable water/cement ratio ranging from 0.443 to 0.557, assuming three intermediate values resulting from the adopted experiment plan (0.46, 0.50, and 0.54) and with a variable proportion of epoxy resin-based powder coating production waste additive ranging from 4 to 11% of the cement mass. Furthermore, two research points were adopted to extend the scope of the experiment in terms of lower values of *w*/*c* and the full range of additive variability (series 10 and 11). Reference concretes without a modifier were made for three *w*/*c* values of 0.443, 0.50, and 0.557, respectively (series A, B, and C).

Based on the adopted experiment plan, the composition of the concrete mix was determined for individual concrete series (Table 2).

For each test point, compressive strength tests were performed on six samples for each concrete series and a statistical analysis of the results obtained was carried out. Statistical analysis included the determination of location and dispersion measures (mean value, standard error of the mean), analysis of variance homogeneity, analysis of the significance of the influence of variables in the experimental design on compressive strength (qualitative analysis), and, in the case of the obtained model, analysis of the significance of effects, analysis of the significance of regression coefficients and analysis of model adequacy.

## 3. Results and Discussion

### 3.1. TGA, DSC and FTIR Analysis of Powder Coating Production Waste

**Figure 5:**
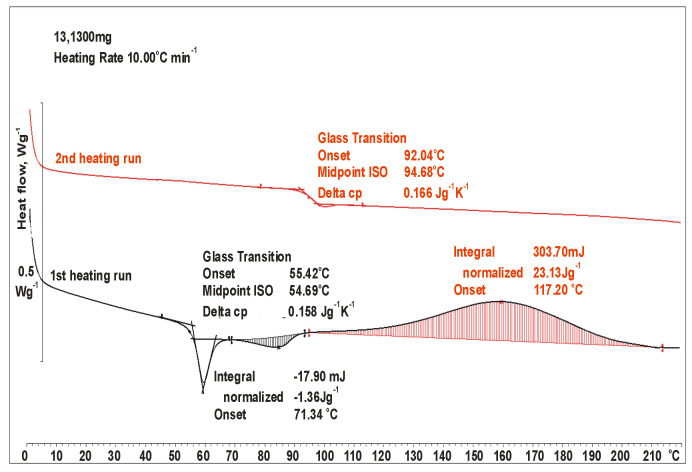
DSC curves of epoxy powder coating waste: black line—1st heating run; red line—2nd heating run.

Figure 5 shows the DSC thermograms for powder coating waste. During the first heating cycle, in the range of 52 to 59 °C, the glass transition at 54.29 °C of the epoxy resin was observed. The observed Tg of the epoxy resin is within the range typical for epoxy powder coatings, e.g., commercial bisphenol A-based epoxy resins E 011 (epoxy number EN = 0.132 mol/100 g) manufactured by Sarzyna Chemical S.A., Nowa Sarzyna, Poland [30]. After exceeding the glass transition temperature (Tg), an endothermic peak was visible in the range of 72–94 °C responsible for the melting of the components of the powder composition. At 100 °C, the cross-linking process begins, which is indicated by the registered exothermic effect. This process occurs over a wide temperature range of 100 to 210 °C, at a heating rate of 10 K/min and with a relatively low exothermic effect (23 J/g). To confirm the complete curing process of the coatings, a second heating cycle was performed. The absence of a signal in the range of 100–210 °C during the second heating cycle indicates the complete occurrence of this process in the first cycle.

**Figure 6:**
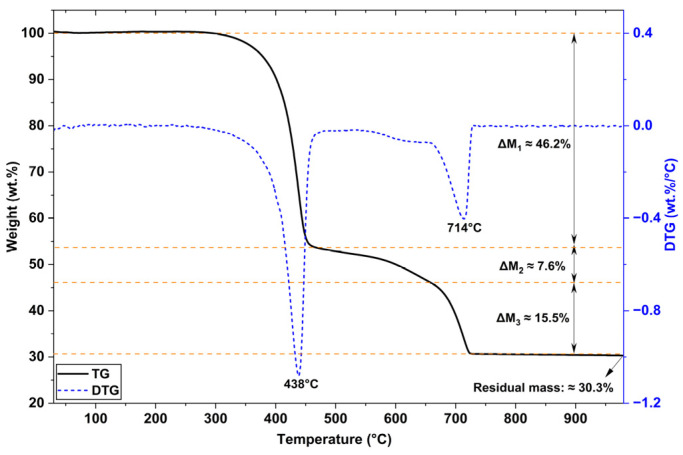
Results of the TGA analysis of the epoxy powder coating waste.

Figure 6 shows the TGA analysis of the waste sample. The first mass loss event (ΔM_1_ ≈ 46%) occurs approximately between 300 °C and 475 °C, with a DTG peak at 438 °C. Taking into account the origin of the waste powder and based on previous results in the literature [31,32], it is possible to suggest that this mass loss in this region was influenced by the thermal degradation of the epoxy resin present in the waste.

The peak of DTG at 714 °C corresponds to a mass loss event that occurred between 650 °C and 740 °C, totaling ΔM3 ≈ 15.5%. Taking into account the presence of calcite (CaCO3) identified by XRD in this study, as well as the characteristic bands associated with the carbonate groups indicated by FTIR, this event can be predominantly attributed to the calcium carbonate decomposition reaction [33]. The residual mass up to 980 °C is approximately 30.3%. Additional tests must be conducted to understand the effect of barite and other phases on the thermal properties of this waste.

The corresponding wavenumber, the possibly associated phases, and the wavenumber of the literature, and the respective references, are presented in Table 3.

**Figure 7:**
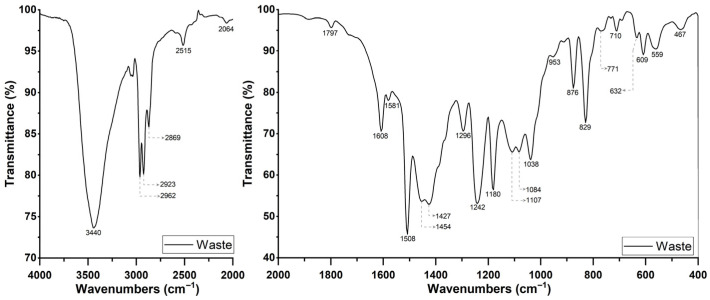
FTIR analysis results of the epoxy powder coating waste figure.

On the basis of the characteristic bands in the FTIR spectrum (Figure 7), it can be concluded that the waste powder used contains epoxy resin, calcite, and barite, which was also confirmed by TGA tests. The signals from the curing agent are not visible. The absence in the IR spectrum of a band at 1700 cm^−1^ resulting from the vibration of carbonyl groups indicates the absence of curing agents containing carboxyl groups. However, the presence of bands at 1508 and 3440 cm^−1^ may indicate the presence of small amounts of curing agents containing amino groups, whose signals are masked by the vibrations of other groups originating from the resin, such as C=C and −OH.

### 3.2. Consistency of the Concrete Mixture

The constant consistency of the concrete mix of 500 ± 20 mm was assumed to be 500 ± 20 mm. For some concrete mixes, the use of a plasticizer was necessary to achieve the desired consistency. The amount of MasterGlenium ACE 560 plasticizer used is shown in Table 1, and the average flow results of the concrete mixture obtained using the flow table method are given in Table 4. No significant deterioration in the consistency of the concrete mix was observed as a result of the addition of epoxy powder coating waste; in fact, a slight improvement in its fluidity was observed.

In terms of consistency testing of the concrete mix, a consistency stability test over time was also carried out with simultaneous reduction in segregation, which is important from the point of view of construction practice. The consistency stability assessment over time was carried out using the slump table method for a series of 9 concrete mixes with a significant proportion of modifiers, corresponding to 7.5% of the cement mass. A slight decrease in the flowability of the concrete mix was observed 30 min after mixing the components, from an initial flow of 485 mm to 470 mm (a decrease of 3%), and after 60 min, a decrease in the initial flow to 450 mm (a decrease of 7.2%). No changes in the homogeneity were observed, nor any segregation of the concrete mixture as a result of the shaking of the table. The segregation of components during the concrete mixture consistency test was only observed 120 min after mixing the components.

### 3.3. Compressive Strength

Compressive strength tests were performed after 28 days of curing the concrete. The results obtained for the average compressive strength together with the standard error of the mean are presented in Table 4. The analysis of variance (ANOVA) performed using Snedecor’s F test (qualitative correlation) showed the statistical significance of the influence of input values (independent variables) on the output value (compressive strength) at a significance level close to zero and at the same time did not confirm the null hypothesis of equal mean values. The Brown–Forsythe homogeneity of variance test showed homogeneity of variance at a significance level of 0.56, which is significantly higher than the accepted 0.05.

Further statistical analysis showed that significant effects at the 0.05 significance level are those related to the linear and quadratic effects of the variable *w*/*c* and the linear effect of the variable *WP*/*c*. The effects related to the interaction between the variables in the plan and the quadratic effect of the *WP*/*c* variable were considered statistically insignificant.

Finally, after checking the significance of the coefficients of the regression equation, model (2) was obtained, allowing the determination of the compressive strength *f_c_* after 28 days of curing concrete modified with waste additives in the form of:

(1) $$f_{c}=472.45-1470.1\cdot w/c+1283.7\cdot {\left(w/c\right)}^{2}-85.2\cdot WP/c,\;R^{2}=0.815$$

where *w*/*c*—water-cement ratio; *WP*/*c*—proportion of waste additive in relation to cement mass.

**Figure 8:**
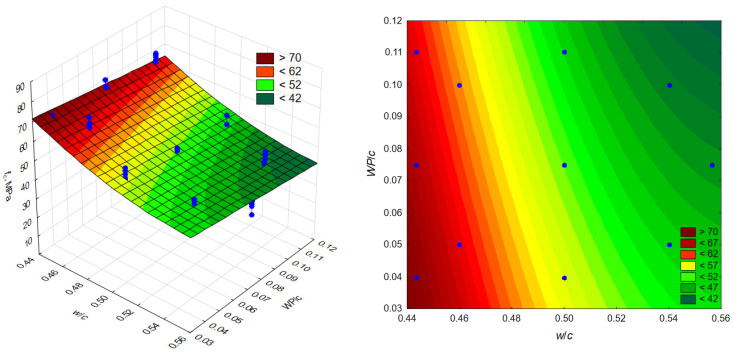
Compressive strength surface and layer graph.

The model obtained (1) is presented graphically in the form of a surface and layer graphs (Figure 8). The obtained value of the determination coefficient R^2^ = 0.815 indicates that the model explains the variability of the compressive strength of concrete modified by changes in *w*/*c* and *WP*/*c* in almost 82%; therefore, 18% is accounted for by other factors, including random factors.

The model accuracy analysis was supplemented with an error analysis and a residual analysis. For the proposed model, the mean square error (MS) was 7.927, while the mean square residual error (MS) was 17.290 (RMSE = 4.17). Analysis of the residual distribution showed that the residual distribution does not deviate significantly from a normal distribution (Kolmogorov–Smirnov test d = 0.0606 and the chi-square test *p* = 0.2354).

The model was validated using compressive strength results for two sets of concrete not included in the experimental plan, with a water-cement ratio of 0.49 and varying amounts of waste additive (5% and 10% of the cement mass). The validation process of the developed regression model confirmed its good predictive ability and reliability in predicting the compressive strength of concrete modified with waste admixture. It should be emphasized that the model can be used to predict compressive strength only within the scope of the experimental study.

The modifier used caused a decrease in compressive strength. However, in the area covered by the experiment, this decrease is most noticeable in the range of high values of *w*/*c* (above 0.5), as evidenced by the changing slope of the layers in the graph (Figure 8). On the other hand, the effect of an increase in the proportion of waste additives on the change in compressive strength of concretes with *w*/*c* in the range of 0.44 to 0.48 is insignificant. The reduction in the adverse effect with increase in the modifier content at low *w*/*c* ratios may result from the sealing effect of the fine-grained additive. In concretes with low *w*/*c* ratios, it is probably necessary to use larger amounts of plasticizer, which promotes the intensification of its interactions with the additive particles.

### 3.4. Density of Concrete

Statistical analysis of the concrete density results obtained after 28 days of cure for all samples within 11 series of modified concretes (66 results) allowed us to obtain a functional relationship with a correlation coefficient R = 0.73 of the form (2):

(2) ρ=2894.0−689.0·w/c−268.5·WP/c

where *w*/*c*—water-cement ratio; *WP*/*c*—proportion of waste additive in relation to cement mass.

**Figure 9:**
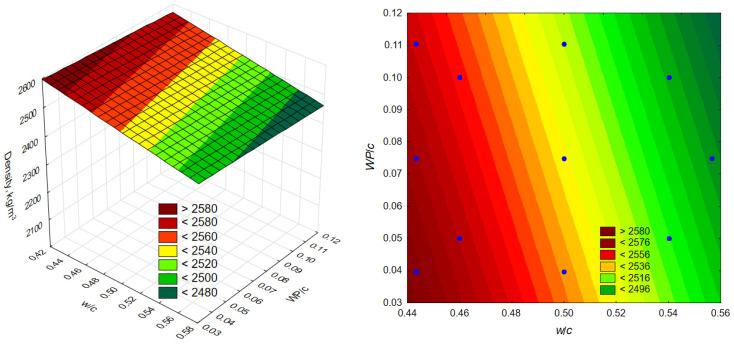
Surface and layer graph of the density of concrete.

The resulting relationship (Equation (2)), presented in a surface and layer diagram (Figure 9), shows the highly significant impact of the *w*/*c* ratio on concrete density, as well as the significant impact of the proportion of waste additive (*WP*/*c*). Due to the linear nature of the influence of both independent variables (*w*/*c*, *WP*/*c*) on the prediction of concrete density, a quantitative assessment of this influence was made on the basis of multiple regression analysis. The influence of the water-cement ratio (*w*/*c*) on the prediction of concrete density was shown to be almost four times greater than the influence of the proportion of waste additive (*WP*/*c*). The significance of the coefficients of the proposed multiple regression equation was also confirmed, as well as the significance of the regression equation, with a significance level close to zero.

Statistical analyses have shown the known effect of changes in the water-cement ratio on concrete density. In the case of higher *w*/*c* ratios of concrete, the increase in its density is mainly due to the presence of chemically bound water, physically adsorbed water, and capillary water. A decrease in the water-cement ratio reduces the water content in concrete also due to a more compact structure and fewer pores, including capillary pores. The changes in concrete density caused by an increase in the proportion of waste additive are consistent throughout the experimental range (Figure 9), which may indirectly indicate that an increase in the amount of additive has no effect on the increase in air content in the concrete mix. The proportional change in density is probably caused by the introduction of the additive as a component of concrete, a component with a much lower density than the other solid components.

The results of the test of the air content in the concrete mix using the pressure method with a porosimeter also confirmed that there was no increase in the porosity of the concrete mix as a result of the addition of waste. In the case of the series 9 mix with *w*/*c* = 0.5 and 7.5% waste additive content, the air content was only 1.5%, which was comparable to the air content of unmodified concrete with the same water-cement ratio.

Any unintentional increase in the air content of the concrete mix would result in a reduction in the durability of the concrete and a deterioration in its strength properties, which would eliminate the possibility of using this modifier.

### 3.5. Environmental Aggression

First, the potential adverse effect of an aggressive corrosive environment on concrete modified with epoxy powder coating waste additives was assessed based on the results of cyclic freeze/thaw resistance tests. Concrete series 9 was tested with a *w*/*c* ratio of 0.5 and a waste additive content of 7.5% by weight of cement mass. The test was carried out to confirm that the modified concrete achieved a frost resistance rating of F150, which, according to the standard, corresponds to between 101 and 150 years of expected service life of the concrete in a structure exposed to frost. This is a typical requirement for structural concrete used in frost conditions, including bridge concrete. The test results confirmed that the modified concrete with waste additive achieved frost resistance class F150. After 150 freeze/thaw cycles, no concrete losses (possible spalling, damaged corners, etc.) were found, no cracks were found on the basis of visual assessment, and the average decrease in compressive strength was 8.9%.

**Figure 10:**
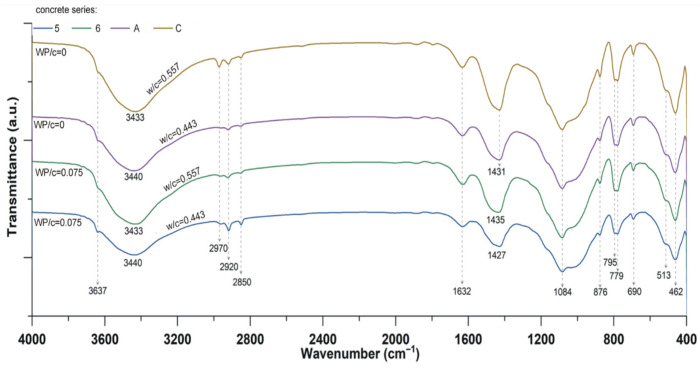
Results of the FTIR analysis of concrete exposed to corrosive solutions.

The impact of an aggressive corrosive environment on concrete modified with waste additives from paint production was also assessed on the basis of tests carried out on modified concrete kept in a corrosive environment of 5% sodium sulfate solution and 5% sodium chloride solution. The study in this area was presented in [42]. As part of this investigation, tests were carried out on the compressive strength of concrete after 90 days of curing, including 62 days of exposure to corrosive solutions. The results of the durability tests showed a slight decrease in the strength of the concrete modified with waste powder compared to the reference concrete. The decrease in strength ranged from 5.4% to 10%. The results obtained indicate that the additive used does not cause significant deterioration in the durability of concrete. At the same time, detailed chemical analyses did not reveal the presence of corrosion reaction products in the concrete. An important supplement to the research on the aggressiveness of corrosive solutions was the FTIR spectrum analysis obtained in the range of 4000–400 cm^−1^. FTIR analysis allowed identification of characteristic bands associated with common phases resulting from hydration of Portland cement (Table 5). By comparing the spectra obtained for concrete modified with waste additives and unmodified concrete, no specific changes related to the introduction of baryte-rich residue were confirmed, neither by band shifts nor by the appearance of new bands (Figure 10). TG and XRD analyses were also performed in this regard.

### 3.6. Potential Reactivity of Waste Coating Powder

To explain the slight negative impact of the waste powder on the change in the compressive strength of concrete, especially in the range of lower *w/c* ratios, the composition of the waste powder and its possible interaction with the concrete components were analyzed. The epoxy resin contained in the powder, due to the presence of reactive epoxy and secondary hydroxyl groups, can react with the plasticizer, which contains carboxyl groups. To confirm whether the aforementioned reaction occurs under concrete hardening conditions, a typical epoxy resin, which is used for the production of powder coatings, was mixed with the plasticizer in a mass ratio of 1:0.25 in a closed polypropylene container and the behavior of the prepared mixture was observed at room temperature. After 24 h, no changes were observed. During the study of the behavior of the mixture at 50 °C, its hardening was observed, indicating the reaction between the resin and the plasticizer [47].

**Figure 11:**
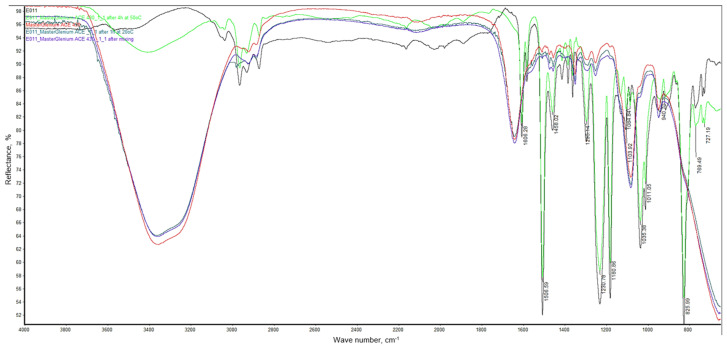
FTIR spectra of epoxy resin E011 (black line), MasterGlenium ACE 560 plasticizer (red line), epoxy resin and plasticizer mixture in a mass ratio of 1:0.25 after mixing (purple line), epoxy resin and plasticizer mixture after 1 h at 20 °C (blue line), epoxy resin and plasticizer mixture after heating during 5 h at 50 °C (green line).

FTIR spectra of mixture taken from the PP container immediately after mixing the ingredients, 1 h after mixing them and after 4 h heating at 50 °C shown on Figure 11.

The spectrum of the mixture recorded after mixing and after 1 h at 20 °C does not differ significantly from the spectrum of the plasticizer, which may indicate that the surface of the resin particles was covered with a plasticizer.

The spectrum of the mixture recorded after 4 h of heating shows a decrease in the intensity of the band at approximately 3450 cm^−1^ originating from the stretching vibrations of the –OH groups of the plasticizer and water, which were present in the plasticizer and partially evaporated, condensing on the walls of the vessel. The intensity of the band at approx. 1650 cm^−1^ coming from the stretching vibrations of C=O, which form the carboxyl group of the plasticizer is also reduced. A small decrease in the intensity of the bands originating from the vibrations of the epoxy ring was also observed at 831 cm^−1^ (symmetric stretching vibration) and at 940 cm^−1^ (asymmetric stretching vibration). A decrease in the intensity of these bands may indicate the reaction of epoxy groups with the carboxyl groups of the plasticizer. To estimate how many epoxy groups reacted with the carboxyl groups of the plasticizer, the change in absorption of these bands was referred to an invariant aromatic C=C peak at ~1600 cm^−1^ of the epoxy resin. For both bands, the absorption changes were approximately 13%, indicating that only a small part of the epoxy groups reacted with the carboxyl groups of the plasticizer. At the same time, the observed significant decrease in the intensity of the band at approximately 1640 cm^−1^, originating from the stretching vibrations of the plasticizer’s carbonyl groups, confirms its reaction with the epoxy resin. As a result of the significant decrease in the intensity of this band, the possible penetration of the plasticizer into the resin grains cannot be ruled out, which was observed as a change in its consistency from a liquid suspension to a solid, hard mass. As a result of these processes, a slight change in the strength of the concrete was observed.

**Figure 12:**
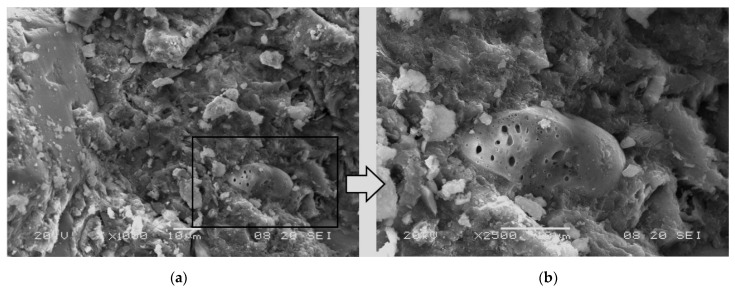
Microstructure of modified concrete series 9 (*w*/*c* = 0.5, 7.5% *WP*); visible microstructure of hardened cement paste with a single grain of waste powder: (**a**) magnification ×1000; (**b**) magnification ×2500.

**Figure 13:**
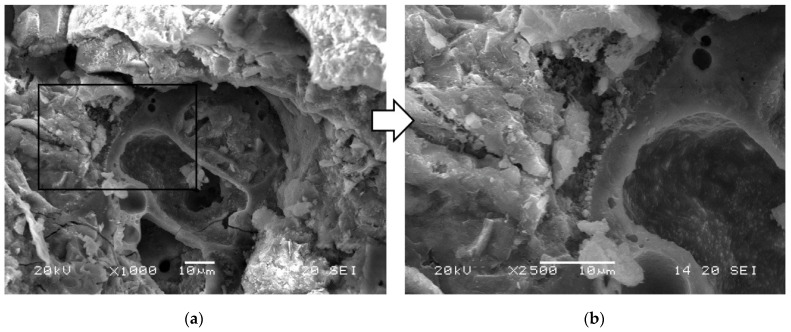
Microstructure of modified concrete series 9 (*w*/*c* = 0.5, 7.5% *WP*); visible microstructure of hardened cement paste with a single grain of waste powder: (**a**) magnification ×1000; (**b**) magnification ×2500.

The absence of any negative impact of the waste powder on the microstructure of the cement matrix was also confirmed by qualitative SEM analyses. In the aggregate grains and the hardened cement paste, good bonding was observed between the aggregate grains and the matrix and a compact microstructure of the hardened cement paste (Figure 12a), as well as a few visible dust grains embedded in the hardened cement paste (Figure 12b). The good bonding of the modifier grains is also confirmed by the observation of a few grains embedded in the cement matrix (Figure 13). The difficulty in identifying fine powdered grains in the analyzed SEM images of concrete fractures may result from their good bonding with the cement matrix and the favorable morphology of the grains, which promotes anchoring in the structure of hardened cement paste and the lack of the tendency to form agglomerations. The absence of deterioration of the contact zone between the matrix and these grains would probably be evident in numerous fractures that run along the phase boundary, such as in concretes containing low adhesion crushed stone aggregate. The visible fracture of the waste powder grain (Figure 13a), which is not filled with hardened cement paste, may confirm the incorporation of this component into the cement matrix. Additionally, no increase in porosity was found at the phase boundary between the cement matrix and the waste powder grain, and no microcracks were found in this area, only numerous cement hydration products that tightly fill the contact zone (Figure 13b).

## 4. Conclusions

On the basis of the results obtained, the following findings and conclusions were reached:

It is possible to use powders obtained from the production of epoxy coatings as an additional component in concrete; however, the effectiveness of this approach depends significantly on the water-cement ratio. Concrete containing 11% waste additive and a water-cement ratio (*w*/*c*) of 0.443 exhibited a compressive strength more than 3% higher than that of the reference concrete without this additive. The observed increase in the compressive strength of the modified concrete can be attributed to the sealing effect of the additive (immediate effect), as well as additional chemical interactions between the component of the mixture, leading to densification of the concrete microstructure. At the same time, at higher *w*/*c* ratios, the additive loses its effectiveness and even impairs its mechanical properties, indicating the existence of an optimal range for its use.

A regression model was proposed that, with sufficient accuracy (R^2^ = 0.815), can be used as a tool for preliminary prediction of the strength of concrete modified with this type of additive. The average agreement between the predictions and the experimental results exceeded 88%.

No significant deterioration was observed in the workability of the concrete mix despite the addition of the waste-based additive. The stability of the properties of the concrete mix over time was demonstrated, as was the absence of any negative impact of the additive on a possible increase in the air content of the concrete mix, which is crucial for the production, transport, and placement of the mix and contributes to its variability.

The proposed modification of the concrete mix does not compromise its durability, indicating that it can be used safely in structural elements. The results confirm the resistance of the modified concrete to environmental factors, including cyclic freezing and thawing (frost resistance class F150) and aggressive environments. The observed resistance to sulfate and chloride corrosion, as well as the absence of significant changes in the structure of the material, indicate that the presence of the additive does not initiate degradation processes in the cement matrix. The limited decrease in compressive strength after exposure to aggressive environments (less than 10%) confirms the stability of mechanical properties under service conditions.

Based on the results of the study, it can be concluded that the residue of epoxy powder coatings can be used as an additive in concrete, provided the mix design is appropriately selected.

## Figures and Tables

**Table 1:** Chemical, physical and mechanical parameters of cement.

Chemical Parameters	Content, % by Mass
SO_3_ sulfate content	2.57
Chloride content Cl^−^	0.03
Alkali content Na_2_O eq	0.65
SiO_2_	19.83
Al_2_O_3_	5.01
Fe_2_O_3_	2.42
CaO	63.29
MgO	1.70
**Mechanical Parameters**	**Reached Values**
Compressive strength, MPa 28 days	55.3

**Table 2:** Composition of concrete mixes for individual series table.

Series	Variables		Concrete Mixture Components per 1 m^3^					
	w/c	WP/c	Cement, kg	Water, dm^3^	Waste Powder, kg	Sand, kg	Basalt Aggregate, kg	SP, % Cement Mass
1	0.460	0.050	368	169.3	18.4	608.5	1367.7	0.45
2	0.460	0.100		169.3	36.8	599.2	1346.7	0.20
3	0.540	0.050		198.7	18.4	583.0	1310.3	-
4	0.540	0.100		198.7	36.8	573.7	1289.3	-
5	0.443	0.075		163.2	27.6	609.2	1369.1	0.50
6	0.557	0.075		204.8	27.6	573.0	1287.9	-
7	0.500	0.040		184.0	14.6	597.7	1343.3	0.20
8	0.500	0.110		184.0	40.6	584.5	1313.6	0.20
9	0.500	0.075		184.0	27.6	591.1	1328.5	0.20
10	0.443	0.040		163.2	14.6	615.8	1383.9	0.80
11	0.443	0.110		163.2	40.6	602.6	1354.2	1.00
A	0.443	-		163.2	-	623.2	1400.6	0.60
B	0.500	-		184.0	-	605.1	1360.0	0.30
C	0.557	-		204.8	-	587.0	1319.3	-

**Table 3:** Summary of the results of the FTIR analysis of epoxy powder coating waste.

Peak Wavenumber Identified	Possible Phase	Assignment	Wavenumber Reference	Reference
cm^−1^	-	-	cm^−1^	-
3440	Water or epoxy resin	O–H	3440	[34]
2962	Epoxy resin	C–H of CH_2_ and CH aromatic and aliphatic	2965–2873	[35]
2923				
2869				
1608		C=C of aromatic ring	1608	
1508		C–C of aromatic	1509	
1427	Calcite	CO_3_^2−^	1420–1490	[36]
1242	Epoxy resin	Ether bonds in epoxide groups	1246	[37]
1180	Barite	SO_4_^2−^	1180	[38]
1107	Barite	SO_4_^2−^	1080–1130	[39]
1084	Barite	SO_4_^2−^	1090	[38]
953	Epoxy resin	C–O–C of oxirane group (asymmetric vibration)	950–810	[35]
876	Calcite	CO_3_^2−^	870	[38]
			880	[40]
829	Epoxy resin	C–O–C of oxirane group (symmetric vibration)	831	[35]
710	Calcite	CO_3_^2−^	714	[36]
			712	[40]
632	Barite	SO_4_^2−^	610–638	[39]
609	Barite			
467	-	Si–O	460	[41]

**Table 4:** Results of the consistency testing of the concrete mix and the compressive strength testing.

Series	Variables		Flow, mm	Compressive Strength, MPa	
	w/c	WP/c		Average, $f_{cm}$	±Standard Error
1	0.460	0.050	505	64.7	1.13
2	0.460	0.100	460	53.7	0.71
3	0.540	0.050	515	50.8	0.37
4	0.540	0.100	500	48.2	1.09
5	0.443	0.075	510	67.4	2.03
6	0.557	0.075	515	41.4	1.17
7	0.500	0.040	500	56.8	0.97
8	0.500	0.110	505	47.2	1.47
9	0.500	0.075	485	51.0	1.23
10	0.443	0.040	475	67.2	0.86
11	0.443	0.110	475	68.4	0.76
A	0.443	-	475	65.3	1.53
B	0.500	-	500	64.3	0.81
C	0.557	-	515	46.9	0.62

**Table 5:** Summary of the results of the FTIR analysis for concrete.

Peak Wavenumber Identified	Possible Phase	Assignment	Wavenumber Reference	Reference
cm^−1^	-	-	cm^−1^	-
3637	Ca(OH)_2_	O–H	3640	[34]
3444–3429	Water	O–H	3440	
1632	Water	H–O–H	1640	[43]
1435–1427	Calcite	CO_3_^2−^	1420–1490	[36]
			1480–1430	[44]
1084	Calcite	CO_3_^2−^	1080	[45]
876	Calcite	CO_3_^2−^	875	[44]
795	Quartz	Si–O	~800	[46]
513	-	Si–O	525	[44]
462	-	Si–O	460	

## Data Availability

The original contributions presented in this study are included in the article. Further inquiries can be directed to the corresponding authors.
